# 

**Published:** 1858-04

**Authors:** 


### THE SANITARY REVIEW.

### CONTENTS OF No. XIII.

The Health of the English Soldier . . . . . . 1
Physiology: its Place in General Education . . . . . 23
Alcohol: its Place and Power . . . . . . 32

**EPITOME OF SANITARY LITERATURE:**

Sanitary Legislation and Administration . . . . . 41
Hygienic Treatment of Epilepsy . . . . . 43
Dr. Inman on the Progress of Rational Medicine . . . 44
Prevention of Diseases of the Skin . . . . . 45
Effect of Occupation on Mortality . . . . . 46
Is the Thames pernicious? . . . . . 47
Miscellaneous Notices . . . . . . 48

**ORIGINAL COMMUNICATIONS:**

Health of the Officers employed in Her Majesty's Post Office during the year 1857. By Waller Lewis, M.B. . . . . 49
Injurious Effects of Underground Kitchens. By F. J. Brown, M.D. . 58
Epidemics and their Everyday Causes. By W. I. Cox, Esq. . . 60
Influence of Sewer Emanations. By T. H. Barker, M.D. . . 70
Scheme for the Drainage of London. By J. E. Huxley, M.D. . 82

**SANITARY AND SOCIAL SCIENCE.**

The Public Health in 1857 . . . . . 86
The Clothing of Soldiers . . . . . 90
London Cow-houses . . . . . . 91
Hygienic Treatment of Pulmonary Consumption . . . 94

**PROGRESS OF EPIDEMICS in Thirty-eight Towns and Districts, contributed specially, by the Staff of Local Observers** . . . . 94

**SANITARY LEGISLATION:**

Public Health Bill . . . . . . 111
Letter from Metropolitan Board of Works to Sir B. Hall . . *ib.*
Reports and Returns . . . . . . 112

**TRANSACTIONS of the EPIDEMIOLOGICAL SOCIETY of LONDON:**

Account of an Epidemic of Small-Pox in Jamaica in 1851-2. By E. C. Seaton, M.D. . . . . . . 1
Report of an Outbreak of Fever among the Crew of the Ottoman line-of-battle ship, Tesherefieh. By J. N. Radcliffe, Esq. . . 13
Quarterly Report of Proceedings . . . . . 20

